# Clinical Effects and Pharmacokinetic Profile of Intramuscular Dexmedetomidine (10 μg/kg) in Cats

**DOI:** 10.3390/ani14152274

**Published:** 2024-08-05

**Authors:** Naftáli S. Fernandes, Yanna D. B. Passos, Kathryn N. Arcoverde, Andressa N. Mouta, Thainá C. Paiva, Kalyne D. S. Oliveira, Gabriel Araujo-Silva, Valéria Veras de Paula

**Affiliations:** 1 Department of Animal Sciences, Semi-Arid Federal University, Mossoró 59625-900, RN, Brazil; nafta_le@hotmail.com (N.S.F.); yannapassosanest@gmail.com (Y.D.B.P.); kathrynnobrega@gmail.com (K.N.A.); andressanmouta@hotmail.com (A.N.M.); thainacacao99@gmail.com (T.C.P.); kalyne_danielly@hotmail.com (K.D.S.O.); 2 Chemistry School, Amapá State University, Macapá 68900-070, AP, Brazil; gabriel.silva@ueap.edu.br

**Keywords:** sedation, alpha-2-adrenergic receptor agonist, pharmacokinetic parameters, feline

## Abstract

**Simple Summary:**

This study investigated how dexmedetomidine, a sedative, works when given to healthy cats via intramuscular injection at a dose of 10 μg/kg. Nine adult cats were observed before and after receiving the drug, with blood samples taken at various times to measure drug levels. Dexmedetomidine acted quickly, causing sedation that lasted for at least an hour, with peak effects between 20 and 30 min and a half-life of about 70 min. Significant decreases in heart rate, respiratory rate, and blood pressure were observed in the first hour, along with an increase in blood sugar levels after 60 min. Overall, dexmedetomidine proved to be effective and safe for sedation in healthy cats, providing valuable information for veterinarians to ensure its safe and effective use in feline patients.

**Abstract:**

This study investigated the pharmacokinetic profile of and pharmacodynamic response to dexmedetomidine administered intramuscularly (IM) at a dose of 10 μg/kg in healthy cats. Nine adult cats were evaluated before and after administration of the drug, with serial collections of plasma samples. Dexmedetomidine induced deep sedation, with a rapid onset of action and a duration of one hour, reaching a peak between 20 and 30 min after administration. The half-life (T½) was 70.2 ± 48 min, with a maximum concentration (Cmax) of 2.2 ± 1.9 ng/mL and time to reach maximum concentration (Tmax) of 26.4 ± 19.8 min. The area under the curve (AUC) was 167.1 ± 149.1 ng/mL*min, with a volume of distribution (Vd) of 2159.9 ± 3237.8 mL/kg and clearance (Cl) of 25.8 ± 33.0 mL/min/kg. There was a reduction in heart rate (HR) and respiratory rate (RR) in relation to the baseline, with a slight decrease in systolic (SBP), diastolic (DBP), and mean (MAP) blood pressure in the first hour. Blood glucose increased after 60 min. Dexmedetomidine proved to be effective and safe, with rapid absorption, metabolization, and elimination, promoting good sedation with minimal adverse effects after IM administration in healthy cats.

## 1. Introduction

Caring for feline patients is a challenge when they present withdrawn and aggressive behavior [1]. In cats that demonstrate these behaviors, when cat-friendly techniques are not sufficient, sedation is commonly practiced in feline clinics. Sedation is often necessary to perform diagnostic tests and for therapeutic management, as well as part of balanced anesthesia protocols [2,3], with the purpose of promoting a reduction in animal stress and ensuring the safety and well-being of the animals and the people who handle them [4].

Drugs from different pharmacological classes are used for sedation in cats. Alpha-2 agonists are the most widely utilized, with dexmedetomidine being the drug of first choice. This drug is licensed in many countries for sedation by intramuscular (IM) administration in cats and has been shown to produce dose-dependent sedation, analgesia, and muscle relaxation. In addition, this route is preferable to other routes of application from the point of view of ease of injection and the prediction of sedative effects [5,6,7]. However, depending on the dose used, these drugs can lead to the appearance of adverse effects, mainly cardiovascular (bradycardia, hypertension, and arrhythmias) and respiratory (hypoventilation), in addition to hyperglycemia. These adverse effects can contraindicate their use, depending on the dose, patient’s physical state, and/or specific situations that may be negatively influenced by the effects mentioned above [8].

Despite the routine use of dexmedetomidine, studies evaluating the clinical effects associated with the pharmacokinetics, in isolation, of doses clinically used in cats via the IM route have not yet been reported. Characterization has been performed in conjunction with other drugs, such as buprenorphine [9] and MK-467 [10], using relatively high doses compared to the doses used on a daily basis. This can lead to inadequate dosage, favoring an increase in the occurrence of adverse effects.

Given this context, the current study aimed to evaluate the clinical effects and pharmacokinetic profile of dexmedetomidine in healthy cats after the administration of 10 µg/kg IM using the liquid chromatography technique combined with mass spectrometry.

## 2. Materials and Methods

### 2.1. Animals

This study was approved by the Animal Use Ethics Committee (21/2023). Nine unneutered male cats were used, with a minimum age of 1 year and a maximum of 5 years, of no defined breed and weighing 3.91 ± 0.71 kg. Research consent was obtained in writing from those responsible for authorization. Admission criteria included vaccinated and dewormed animals and the absence of pharmacological treatment for at least 15 days. The animals underwent physical and laboratory exams, such as blood count, urea, creatinine, alanine aminotransferase (ALT), gamma-glutamyltransferase (GGT), albumin, globulins, total proteins, and electrocardiography. Any alterations in the aforementioned exams resulted in the animals being excluded from this study.

### 2.2. Preparation of Patients

A six-hour food and two-hour water fast was established for all cats. Before the study, patients were anesthetized to place an 18 g catheter in one of the jugular veins, coupled to a PRN adapter plug, in order to collect blood samples for pharmacokinetic analysis. Anesthesia was performed with isoflurane, with induction carried out in a closed acrylic box coupled to the common gas outlet of an inhalation anesthesia device with a universal vaporizer without a flowmeter, with isoflurane diluted in 100% oxygen being supplied at a flow of 4 L/min until the animal reached the anesthetic level that allowed orotracheal intubation using a tracheal tube (with cuff) of the appropriate size for each cat. After intubation, the patient was connected to an anesthesia circuit without a carbon dioxide (CO_2_) absorber, and anesthesia was maintained with isoflurane diluted in 100% oxygen at a flow of 300 mL/kg/min. After obtaining venous access, the supply of the inhalational anesthetic agent was stopped, and after the patient showed an extubation reflex, they were taken to the cattery for complete recovery. To ensure complete elimination of the inhalation agent, a minimum interval of 90 min was given after anesthetic recovery to begin the study.

### 2.3. Drug Administration and Sample Collection

**Figure 1:**
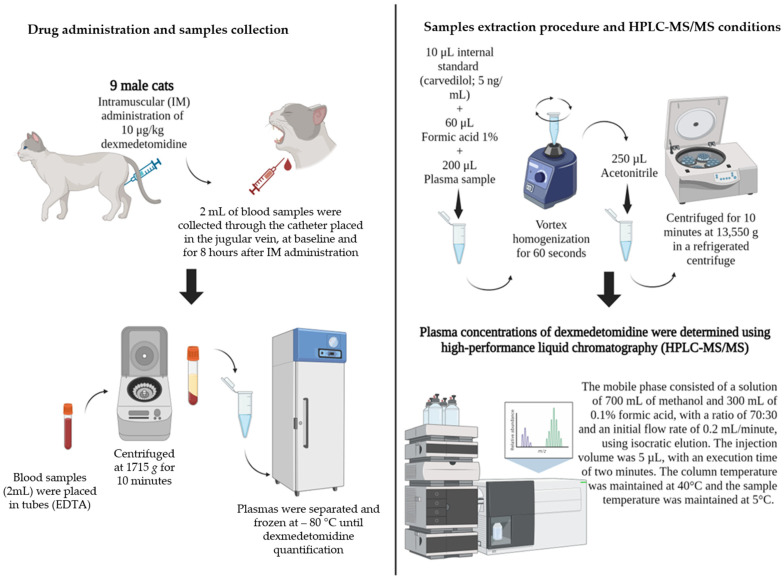
Drug administration, sample collection, extraction procedure, and HPLC-MS/MS conditions in 9 cats after IM administration of 10 µg/kg of dexmedetomidine. Created with BioRender.com.

The pharmacokinetic and pharmacodynamic study was exploratory and descriptive. The treatment consisted of the administration of dexmedetomidine (Dexdomitor, Zoetis, São Paulo, Brazil), administered intramuscularly into the semimembranosus muscle (right or left pelvic limb) at a dose of 10 μg/kg. Treatment administration was carried out by the same investigator (NSF). Blood (2 mL) was collected through the catheter placed in the jugular vein at baseline (0) and 3, 6, 10, 15, 20, 30, 45, 60, 120, 240, and 480 min after intramuscular administration, totaling 24 mL. Blood samples were placed in tubes containing ethylenediaminetetraacetic acid (EDTA), stored on ice, and centrifuged within 1 h of collection at 1715× *g* for 10 min. Plasma was separated and frozen at −80 °C until dexmedetomidine quantification (Figure 1).

### 2.4. Sedation, Physiological Parameters, and Glycemia

Pharmacodynamic assessments were recorded before dexmedetomidine administration and immediately after each blood collection time point. Firstly, sedation was assessed based on the Feline Multiparametric Sedation Score (FMSS) [11], with a score ranging from 0 to 12. Scores of 0 to 2 indicated no sedation; from 3 to 6, light; from 7 to 9, moderate; and from 10 to 12, deep. Subsequently, physiological parameters were evaluated, such as heart rate (HR), obtained through cardiac auscultation for one minute; respiratory frequency (RR), assessed by counting chest excursions for one minute; systolic, diastolic, and mean arterial pressure (SBP, DBP, and MAP), measured using a digital oscillometric method, with the aid of a multiparametric monitor (SDA 8, SDAmed, São Paulo, Brazil); and rectal temperature (RT), using a digital thermometer inserted into the rectum. During blood collection at baseline and 15 and 60 min, a small aliquot of blood (0.1 mL) was used to measure blood glucose using a portable glucometer (OneTouch^®^). The occurrence of emesis, salivation, and nausea at the evaluated moments was also recorded.

### 2.5. Sample Extraction Procedure, Instrumentation, and HPLC-MS/MS Conditions

In total, 10 µL of an internal standard solution [carvedilol (5 ng/mL), Sigma-Aldrich, São Paulo, Brazil] and 60 µL of a 1% formic acid solution in water were added to aliquots of plasma samples (200 µL), followed by vortex homogenization for 60 s. Additionally, 250 µL of acetonitrile was added, and the samples were centrifuged for 10 min at 13,550× *g* in a refrigerated centrifuge. A 5.0 µL aliquot was used for analysis (Figure 1).

Plasma concentrations of dexmedetomidine were determined using high-performance liquid chromatography (HPLC-MS/MS), Nexera X2 UPLC (Shimadzu, Japan), coupled to mass spectrometry, LCMS-8040 (Shimadzu, Japan), using a 1.9 µm reverse-phase Pinnacle DB BiPh column (Phenyl; 2.1 × 50 mm particle size and 1.9 µm; 100 Å pore size) (Restek^®^, Bellefonte, Centre County, PA, USA). The mobile phase consisted of a solution of 700 mL of methanol and 300 mL of 0.1% formic acid, with a ratio of 70:30 and an initial flow rate of 0.2 mL/min, using isocratic elution. The injection volume was 5 µL, with an execution time of two minutes. The column temperature was maintained at 40 °C, and the sample temperature was maintained at 5 °C. The mass spectrometer was set in multiple reaction monitoring (MRM) mode in positive ionization mode (ESI) for dexmedetomidine. The collision energy and cone voltage were 12 and 19 V, respectively. The flow rates of the cone and desolvation gas were calibrated to 150 and 600 L/min, respectively, using argon as the colliding gas at a flow rate of 0.15 mL/min. The mass/charge ratio (*m*/*z*) for dexmedetomidine was 201.1 > 95.0. The standard internal ion (carvedilol/IS) ratio was 407.1 > 366.2. The serial dilutions to prepare the standard curve had concentrations of 0.001, 0.005, 0.25, 1, 5, 10, and 25 ng/mL, and quality controls were 0.5, 2.5, and 17.5 ng/mL. The accuracy and precision of the assay were determined in replicates of 5 at each concentration.

### 2.6. Pharmacokinetic Analysis

Dexmedetomidine’s pharmacokinetic parameters were calculated using a non-compartmental approach using PK Solver 2.0 software (TongJiaXiang, Gulou District, Nanjing, China). The variables obtained were K_a_ (absorption coefficient), λz (elimination constant), T_½_ (half-life time), T_max_ (time to reach maximum concentration or peak time), C_max_ (concentration maximum), AUC_0-t_ (area under the curve from time 0 to the last measurement), AUC_0-inf_ (area under the curve from 0 to infinity), AUC_0-t_/AUC_0-inf_ (ratio between AUC0-t and AUCinf), Vd (volume of distribution), and Cl (total clearance).

### 2.7. Statistical Analysis

Data are expressed as mean ± standard deviation, median, standard error, and minimum and maximum using the statistical program SAS v8 (System for Windows—SAS Institute, Cary, NC, USA). After checking the parametric assumptions for each variable studied, the statistical differences between the times studied in relation to the baseline were analyzed. When the data were parametric, they were verified through analysis using a mixed-effect model for repeated measures (Proc Mixed from the SAS program), followed by the Dunnett test. Data that did not present a normal distribution even after transformation were considered non-parametric and compared using the Friedman test. The significance level established was 5%.

## 3. Results

**Figure 2:**
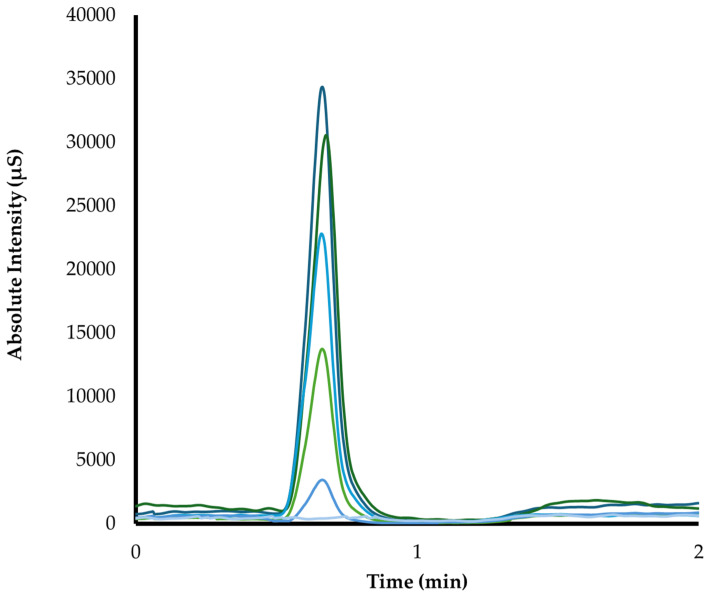
Schematic chromatogram obtained by HPLC-MS/MS showing the bioanalytical curves of dexamethasone (DEX), a blank, and five calibration points (0.25–25 ng/mL). The mass/charge ratio (*m*/*z*) for dexmedetomidine was 201.1 > 95.0.

The method was developed and validated. Figure 2 shows the DEX schematic chromatogram of the calibration curve (five points). The accuracy values at 0.5, 2.5, and 17.5 ng/mL were 8.27%, 10.9%, and 14.38%, respectively. The precision values (coefficients of variation) at 0.5, 2.5, and 17.5 ng/mL were 2%, 2%, and 1%, respectively. The detection level was <0.001 ng/mL (signal/noise = 47 to 1 pg/mL), but the precision was not greater than ±20%. The quantification level was 0.25 ng/mL (signal/noise = 96) with a precision less than or equal to ±15%.

**Figure 3:**
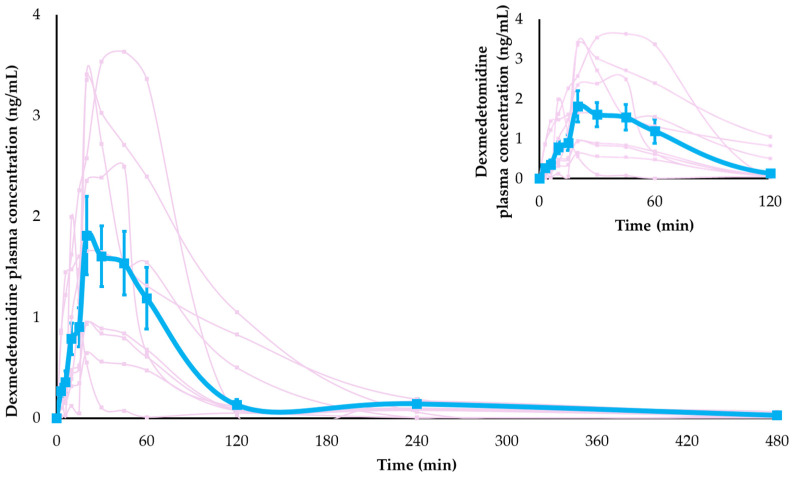
The plasma concentration profiles of dexmedetomidine over 8 h after IM administration of 10 µg/kg of dexmedetomidine in all nine cats are shown in pink lines. The mean plasma concentration is represented by a blue line. Data are expressed as mean ± SD. The values observed between the 0–120-min time points are highlighted.

Dexmedetomidine was detectable in plasma within 3 min after the injection of a dosage of 10 µg/kg into semi-membranous muscles, with measurable concentrations for up to 480 min (Figure 3).

The non-compartmental model best described the pharmacokinetic parameters of dexmedetomidine, with a C_max_ of 2.2 ± 1.9 ng/mL, T_max_ of 26.4 ± 18.6 min, and T_½_ of 70.2 ± 48 min. The other pharmacokinetic parameters are described in Table 1.

The bioavailability (F) of 10 µg/kg dexmedetomidine administered via the IM route in this study was calculated based on the equation [(AUC_0-t_/IM dose ratio)/(AUC_0-t_/IV dose ratio) × 100], using the AUC_0-t_/IV dose ratio from studies published by other authors since dexmedetomidine was administered exclusively via the IM route. The F values are shown in Table 2.

**Figure 4:**
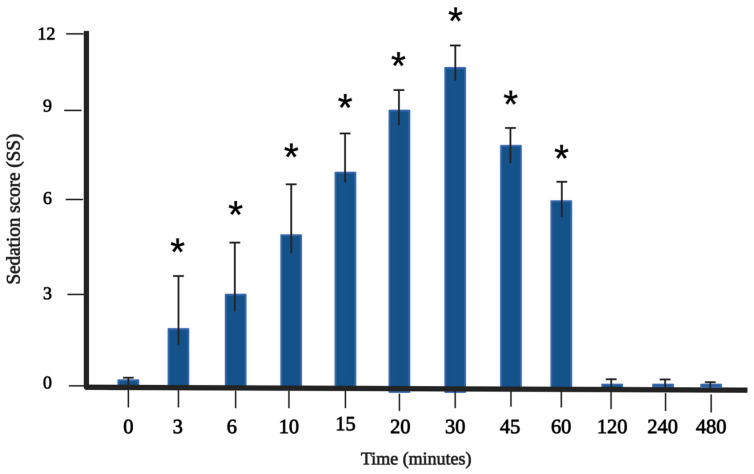
Sedation scores obtained up to 480 min after intramuscular administration of 10 μg/kg (IM) of dexmedetomidine in nine healthy cats. Data expressed as median and standard error. * Indicates statistically significant differences between baseline and corresponding time point (*p* < 0.05 Friedman). Created with BioRender.com.

Regarding the sedative effect, before the application of dexmedetomidine, none of the cats showed behavior compatible with any condition that would score a level of sedation, as assessed by the Feline Multiparametric Sedation Score (FMSS) [11], as can be seen in Figure 4 and Table 3.

**Figure 5:**
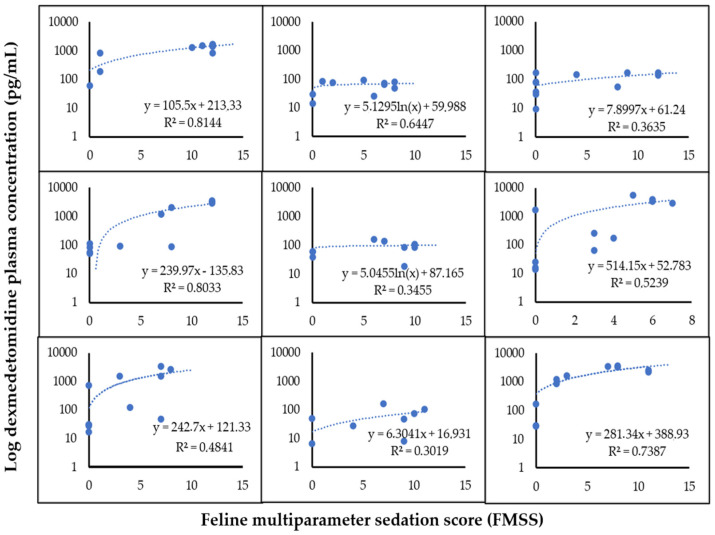
PK/PD model for correlation between plasma concentration and Feline Multiparametric Sedation Score (FMSS) of 9 healthy cats after IM administration of 10 µg/kg of dexmedetomidine.

When analyzing the PK/PD model for the correlation between the plasma concentration and its sedative effects after IM administration of dexmedetomidine individually in the animals in this study, a positive linear correlation was verified in 77.7% of the cats (n = 7), and a logarithmic correlation was observed in 22.3% (n = 2) of the animals (Figure 5).

**Figure 6:**
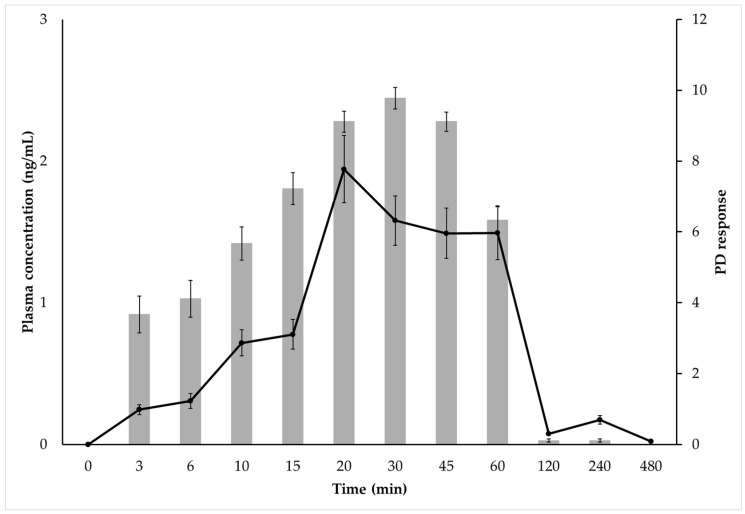
Relationship between plasma concentrations and sedation scores (SSs) of 9 healthy cats after IM administration of 10 µg/kg of dexmedetomidine.

The graphical representation of the mean plasma concentration and mean sedation score over the observed period (Figure 6) showed a delay in the maximum sedation score (30 min) compared to the peak plasma concentration of dexmedetomidine (20 min).

Regarding the effects on cardiorespiratory parameters, a decrease was observed after the administration of dexmedetomidine, as shown in Table 4.

A significant reduction in heart rate occurred 3 min after administration and was maintained up to 2 h after application, with the lowest average at 45 min in relation to the baseline value. The mean respiratory rate differed significantly from the baseline after 15 min of assessment, remaining statistically lower up to 1 h after administration. With regard to systolic blood pressure, a significant reduction occurred between 10 and 60 min after application, reaching the lowest average at 60 min in relation to the baseline value. Mean and diastolic blood pressure values showed a statistical reduction only 60 min after application when compared to baseline values. No difference was observed in rectal temperature values throughout the study period.

Regarding glycemia, a significant increase in blood glucose levels was observed 1 h after IM administration when compared to the baseline (Table 3). None of the animals presented sialorrhea. However, 55.5% of cats (five animals) presented episodes of vomiting within the first 20 min after the application of dexmedetomidine: one cat with one episode, three cats with two episodes and one cat with three episodes of emesis. There were no serious adverse effects with the use of dexmedetomidine in this study, and the sedative effects were directly proportional to plasma concentrations.

## 4. Discussion

This is the first study to describe the pharmacokinetic profile of and pharmacodynamic response to intramuscular dexmedetomidine (10 μg/Kg) in cats. Despite its routine use, few studies have characterized the pharmacokinetic parameters of dexmedetomidine administered via the IM route in cats [9,10]. The research carried out to date has mainly used the intravenous (IV) or oral transmucosal routes and in doses higher than those currently used in this species via the intramuscular route [9,12,13,14,15,16].

The administration of 10 μg/kg of dexmedetomidine via the IM route showed pharmacokinetic behavior similar to that reported in studies with dogs using 10 μg/kg and in cats using 25 μg/kg via the IM route, in which one application promoted rapid absorption and a short elimination half-life [10,17,18]. The time until reaching the maximum concentration of dexmedetomidine in the current study was close to that observed after an IM injection of 25 μg/kg alone (17.8 min) and 40 μg/kg associated with buprenorphine (18 min) in cats [9,10].

The mean peak plasma concentration in the current study occurred 10 min before the maximum peak of sedation. This effect can occur with drugs that act directly on the central nervous system (CNS) due to a delay in distribution to the site of action and indirect mechanisms of action, such as uptake at the active site, slow receptor kinetics, delayed or modified activity, and time-dependent protein binding, among other factors [19]. The time to reach peak sedation after IM administration of 10 µg/kg was consistent with values reported using the same dose in dogs, alone or associated with methadone, at 30 min after IM administration [17,18]. The C_max_ of 2.2 ng/mL observed in this study was slightly different from that observed with doses of 25 and 40 μg/kg IM, alone or associated with buprenorphine in cats (10.2 ng/mL and 22.07 ng/mL, respectively) [9,10]. After adjusting the maximum concentration (C_max_) for the dose administered, it was found that the concentrations obtained in the different studies were comparable. In the current study, the adjusted concentration reached 220 pg/mL/µg/kg. For doses of 25 and 40 µg/kg, the adjusted concentrations were 408 and 552 pg/mL/µg/kg, respectively. This is because the dose administered in this study is lower than those used in previous studies. The maximum concentration (C_max_) is directly corelated to the dose administered (y = 11x + 117; R^2^ = 0.99) and the possible activation and no saturation of metabolic pathways [20].

Although it is commonly administered via the IM route, the bioavailability of dexmedetomidine in cats has only been reported by a single study [10], in which a bioavailability of 52% was observed after an IM injection of 25 μg/kg. Bioavailability was not obtained in the current study since a pharmacokinetic analysis was not performed intravenously, making it impossible to calculate the actual bioavailability in the cats in this research. However, using the AUC/dose ratios measured after IV administration in studies published by other researchers, bioavailability was estimated to be between 15 and 30% (Table 2) [10,12,13,14]. Despite the lower bioavailability comparated to the study cited above [10], it is important to emphasize that some of the data were obtained from previously published studies by different researchers, which implies methodological variability and differences in the samples, animals and breeds studied, which may significantly alter the disposition of the drug in question in different organisms.

**Figure 7:**
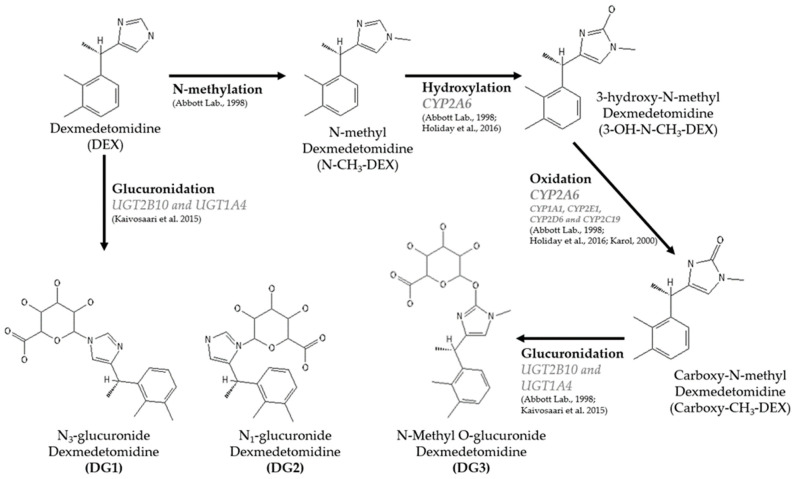
Liver metabolism of dexmedetomidine [21,22,23,24,25].

The pharmacokinetic results indicate rapid metabolization, with a gradual reduction in plasma concentration after an intramuscular injection of 10 μg/kg of dexmedetomidine. The calculated T_½_ was approximately 70.2 ± 48 min, similar to values observed when using 25 μg/kg alone (62.3 min) [10] and 40 μg/kg when associated with buprenorphine after IM application (76.2 min) [9], showing similar clearance at different doses. This effect was observed after IV administration of a bolus of three different doses (5, 20, and 50 µg/kg) in cats, characterized by moderate clearance with minimal dose dependence [13]. The metabolism of dexmedetomidine involves several pathways and mainly occurs in the liver [21]. Initially, dexmedetomidine undergoes N-methylation to form N-methyl dexmedeto-mi-dine (N-CH_3_-DEX) [21]. N-CH_3_-DEX can then be hydroxylated by the enzyme CYP2A6 to produce 3-hydroxy-N-methyl dexmedetomidine (3-OH-N-CH_3_-DEX) [22]. This metabolite undergoes further oxidation by CYP2A6 and other CYP450 enzymes, such as CYP1A1, CYP2E1, CYP2D6, and CYP2C19, resulting in carboxy-N-methyl dex-me-detomidine (Carboxy-CH_3_-DEX). Additionally, DEX and its metabolites can be glucuronidated. Specifically, DEX is directly glucuronidated by UGT2B10 and UGT1A4 to form N_3_-glucuronide dexmedetomidine (DG1) and N_1_-glucuronide dexmedetomidine (DG2), with UGT2B10 playing a significant role in the glucuronidation process. This comprehensive metabolic pathway highlights the extensive biotransformation of dexmedetomidine, primarily through N-glucuronidation and oxidation, leading to inactive metabolites that are excreted predominantly in the urine (Figure 7) [23,24,25].

A deficiency in the glucuronization of drugs in cats was commented on in scientific circles a few years ago. However, it has already been determined that this deficiency does not apply to all medications that undergo glucuronization but rather depends on the structure of the drug, such as the phenolic structure in its composition [26]. Unlike in humans and dogs, the liver of a cat does not express UGT1A4 but does express UGT1A1 and UGT1A2. These two isoforms are possibly responsible for the hepatic metabolism of dexmedetomidine in feline species [26,27]. However, more studies are needed to identify metabolites and possible metabolic pathways in cats. Despite these particularities, the half-life in the animals in this study is similar to that described for the drug in dogs and humans, with a half-life of less than 2 h [20,23,28,29].

The sedation obtained after an IM injection of 10 μg/kg dexmedetomidine was satisfactory. The sedative effects of a 10 μg/kg IM dose of dexmedetomidine, alone or associated with opioids or dissociative agents, have already been investigated in cats and dogs, promoting sedation with a rapid onset of action and a duration of more than 30 min [2,17,18,30], as observed in the cats in the current research. The sedative effects of a dose of 20 μg/kg were similar to those of a dose of 10 μg/kg associated with pethidine or butorphanol after IM application in cats [2]. However, 10 μg/kg of dexmedetomidine alone provided less sedation than the combination with butorphanol or ketamine via the IM route [30].

In the patients in the current study, HR and RR were inversely correlated with the plasma concentration. Similar behavior was also observed in dogs using the same dose via the IM route [17]. A reduction in heart rate was also reported by other authors after IM administration of dexmedetomidine, alone or associated with opioids, in cats [5,30,31]. In cats, dexmedetomidine promotes a decrease in heart rate similar to that caused by medetomidine, resulting from a decrease in central sympathetic activity, leading to an increase in parasympathetic tone [31,32,33,34]. The respiratory effects caused by the administration of dexmedetomidine are mild, with a decrease in RR and minute volume [35]. In the current study, RR significantly decreased in relation to the baseline at times when plasma concentrations were highest (between 15 and 60 min), suggesting a direct effect of the plasma concentration on this clinical parameter. A reduction in RR was also present after the use of dexmedetomidine via the IM route, either alone or in combination, in cats and dogs [5,17,18,30,31,36,37]. Low partial oxygen saturation values (SpO_2_), RR, partial pressure of arterial oxygen (PaO_2_), and high arterial carbon dioxide partial pressure values (PaCO_2_) were observed, indicating moderate hypoxemia and respiratory depression after the injection of a combination of dexmedetomidine, methadone, and midazolam in cats [38]. Despite these findings, it is not possible to state that respiratory depression occurred, since SpO_2_ parameters and arterial blood gas measurements were not evaluated. The decrease in RR in the cats in the current research can be attributed to a decrease in basal metabolism caused by sedation [28], which was at its highest levels at these respective moments.

Despite the decreases in SBP, DBP, and MAP in patients in relation to the baseline, at some assessment moments, these decreases were not clinically important, as the values remained within the limits that characterize normotension in cats, with no need for therapeutic intervention with the use of a reverser or vasopressor therapy. Commonly, alpha-2 agonists induce a transient hypertensive condition due to the activation of alpha-1 and alpha-2 receptors present in the vascular endothelium, producing vasoconstriction [39]. This phenomenon was observed after the application of 25 μg/kg IM in cats [31] and after 10 μg/kg associated with methadone via the IM route in dogs [18]. However, results similar to those observed in the cats in the current research are in agreement when administering 10 μg/kg alone and 20 μg/kg associated with buprenorphine IM, where there was a slight reduction in or maintenance of blood pressure [5,30]. These differences highlight the dose-dependent effects of dexmedetomidine on blood pressure, where lower doses lead to smaller changes in blood pressure [40]. Furthermore, the administration route influences the effects of dexmedetomidine, where absorption and distribution after extravascular administration are slower compared to the IV route; so, the IM route can delay the diffusion of the drug to the vascular endothelium, thus reducing its effects on the cardiovascular system [39], as observed in the current study.

The RT did not demonstrate significant changes throughout the observation period, as reported in a previous study, where the same IM dose was used in cats [18]. However, changes in temperature were reported in other studies using dexmedetomidine alone or associated with butorphanol or ketamine. The authors attributed these results to the lack of temperature support and a greater degree of muscle relaxation obtained with the associations described above; it is strongly recommended that body temperature be monitored and maintained in cats, as low temperatures can contribute to an extension of recovery time if a pharmacological reversal agent is not used [2,30,31,36].

Alpha-2 agonists have direct effects on glucose homeostasis, as when binding to alpha-2 receptors, they lead to an increase in blood glucose concentrations in dogs and cats [41,42,43,44]. The increase in glycemia that occurred 60 min after the application of dexmedetomidine in the animals in the current research was also observed when this drug was administered at a dose of 10 μg/kg IV in healthy cats due to the activation of alpha-2 receptors located in pancreatic beta cells, which culminates in the inhibition of insulin release and consequent hyperglycemia [44].

No adverse effects related to the volume of blood collected were observed. The occurrence of adverse effects was practically non-existent, confirming the efficacy and safety of the dose studied. Despite this, episodes of vomiting were recorded, even with prior fasting. Emesis has been reported by other authors after the administration of dexmedetomidine in cats [2,5,36,37,45]. This effect may occur, especially when it is administered in isolation, due to the direct stimulation of the chemoreceptor trigger zone [3]. The sample size in the present study was small (n = 9), and all cats were healthy, young, nonspayed adult males. Therefore, the results of this study may not be representative of the entire cat population or those with specific health conditions.

## 5. Conclusions

Dexmedetomidine at a dose of 10 μg/kg administered intramuscularly promoted deep sedation with a rapid onset of action, a duration greater than 60 min, few alterations in physiological parameters, and no important clinical repercussions. The pharmacokinetic profile was characterized by rapid absorption, metabolization, and elimination in healthy cats, indicating that this drug is a safe alternative for sedation protocols in the species in question.

## Figures and Tables

**Table 1:** Parameters of the pharmacokinetic profile of dexmedetomidine using a non-compartmental model of 9 cats after IM administration of 10 µg/kg of dexmedetomidine.

Parameter	Unit	Mean	Standard Deviation	Standard Error
K_a_	ng/min	5.65	0.58	0.19
λz (Lambda-z)	1/h	0.89	0.62	0.08
T_½_	min	70.2	48.0	6.0
T_max_	min	26.4	18.6	2.4
C_max_	ng/mL	2.2	1.9	0.23
AUC_0-t_	ng/mL*min	167.1	149.2	18.6
AUC_0-inf_	ng/mL*min	169.2	150.7	18.8
AUC_0-t_/AUC_0-inf_		0.99	0.03	0
AUC_0-t_/dose	ng/mL*min/µg/kg	16.71	14.92	1.86
Cmax/dose	ng/mL/µg/kg	0.22	0.19	0.023
Vd	mL/kg	2159.9	3237.8	404.7
Cl	mL/min/kg	25.8	33.0	4.1

K_a_: absorption coefficient; λz: elimination constant; T_½_: half-life time; T_max_: time to reach maximum concentration or peak time; C_max_: maximum concentration; AUC_0-t_: area under the curve from time 0 to the final measurement; AUC_0-inf f_: area under the curve from 0 to infinity; AUC_0-t_/AUC_0-inf_: ratio between AUC_0-t_ and AUC_0-inf_; Vd: distribution volume; Cl: clearance.

**Table 2:** Bioavailability using a non-compartmental model of 9 cats after IM administration of 10 µg/kg of dexmedetomidine. The data obtained were compared with results from previous studies conducted by other authors.

Authors	Dose (µg/kg)	Route	AUC (ng/mL*min)	Ratio AUC/Dose (ng/mL*min/µg/kg)	F (%)
This study	10	IM	167.1	16.7	NA
Escobar et al., 2012 [12]	10	IV	1061	106.1	15.7
Pypendop et al., 2014 [13]	5	IV	542	108.4	15.4
Pypendop et al., 2014 [13]	20	IV	1558	77.9	21.4
Pypendop et al., 2014 [13]	50	IV	3606	72.1	23.2
Pypendop et al., 2016 [14]	12.5	IV	858	68.6	24.3
Pypendop et al., 2016 [14]	25	IV	1382	55.3	30.2
Pypendop et al., 2017 [10]	25	IM	2257	90.3	18.5

IM: intramuscular; IV: intravenous; AUC: area under the curve; F: bioavailability; NA: not applicable.

**Table 3:** Medians, standard errors, and minimum and maximum values of sedation Feline Multiparametric Sedation Score (FMSS) of 9 healthy cats after IM administration of 10 µg/kg of dexmedetomidine.

Time	Median FMSS	Standard Error	Minimum–Maximum
Basal (0)	0 ^A^	0	0–0
3 min	2 ^B^	1.4	0–12
6 min	3 ^B^	1.4	0–12
10 min	5 ^B^	1.3	0–12
15 min	7 ^B^	1.2	0–12
20 min	9 ^B^	0.8	5–12
30 min	11 ^B^	0.8	7–12
45 min	8 ^B^	0.7	6–12
60 min	6 ^B^	1.1	0–10
120 min	0 ^A^	0.1	0–1
240 min	0 ^A^	0.1	0–1
480 min	0 ^A^	0	0–0

^A,B^ Different capital letters in the column mean statistical difference (*p* < 0.05 Friedman).

**Table 4:** Means ± standard deviation of physiological parameters and blood glucose levels of 9 cats after IM administration of 10 µg/kg of dexmedetomidine.

Time	HR ^†^	RR	SBP ^†^	DBP	MAP	RT	Blood Glucose
	(bpm)	(rmpm)	(mmHg)	(mmHg)	(mmHg)	(°C)	(mg/dL)
Basal (0)	199.8 ± 22 ^A^	58.2 ± 13.0 ^A^	156.2 ± 33.4 ^A^	125.3 ± 39.3 ^A^	138.7 ± 34.2 ^A^	38.4 ± 0.6 ^A^	72.1 ± 13.1 ^B^
3 min	126.6 ± 36.8 ^B^	59.1 ± 19.4 ^A^	146.5 ± 50.0 ^A^	118.4 ± 51.3 ^A^	128.5 ± 49.5 ^A^	38.5 ± 0.8 ^A^	-
6 min	112.0 ± 17.4 ^B^	45.8 ± 10.4 ^A^	161.0 ± 37.6 ^A^	134.1 ± 45.8 ^A^	137.0 ± 37.7 ^A^	38.5 ± 0.8 ^A^	-
10 min	127.9 ± 22.7 ^B^	47.2 ± 11.7 ^A^	142.1 ± 25.0 ^B^	112.5 ± 28.5 ^A^	122.3 ± 25.1 ^A^	38.6 ± 0.6 ^A^	-
15 min	115.3 ± 23.8 ^B^	42.9 ± 7.2 ^B^	148.4 ± 23.7 ^B^	111.8 ± 27.1 ^A^	133.3 ± 25.7 ^A^	38.7 ± 0.7 ^A^	110.6 ± 38.1 ^B^
20 min	98.0 ± 15.3 ^B^	44.4 ± 10.9 ^B^	145.3 ± 20.1 ^B^	112.9 ± 28.9 ^A^	131.3 ± 36.7 ^A^	38.8 ± 0.6 ^A^	-
30 min	91.6 ± 18.4 ^B^	40.9 ± 5.6 ^B^	142.0 ± 22.5 ^B^	98.3 ± 25.4 ^A^	113.9 ± 24.2 ^A^	38.6 ± 0.62 ^A^	-
45 min	88.11 ± 12.1 ^B^	33.3 ± 7.2 ^B^	137.7 ± 34.1 ^B^	99.2 ± 32.3 ^A^	114.0 ± 33.2 ^A^	38.2 ± 0.5 ^A^	-
60 min	97.8 ± 37.7 ^B^	35.6 ± 7.6 ^B^	124.3 ± 19.3 ^B^	83.8 ± 20.6 ^B^	98.4 ± 16.6 ^B^	38.2 ± 0.6 ^A^	214.0 ± 76.9 ^A^
120 min	142.9 ± 55.5 ^B^	46.0 ± 17.9 ^A^	134.5 ± 26.4 ^A^	111.0 ± 24.4 ^A^	119.1 ± 24.3 ^A^	37.9 ± 0.8 ^A^	-
240 min	154.8 ± 60.3 ^A^	54.6 ± 12.9 ^A^	147.4 ± 37.1 ^A^	113.8 ± 41.2 ^A^	122.1 ± 41.4 ^A^	38.5 ± 0.6 ^A^	-
480 min	199.8 ± 21.9 ^A^	58.2 ± 13.0 ^A^	164.9 ± 42.0 ^A^	134.2 ± 41.9 ^A^	141.9 ± 43.4 ^A^	38.4 ± 0.6 ^A^	-

^A,B^ Different letters in the column mean statistical difference (*p* < 0.05—Dunnett); † Friedman; SD = standard deviation; HR = heart rate; RR = respiratory rate; SBP = systolic blood pressure; DBP = diastolic blood pressure; MAP = mean arterial pressure; RT: rectal temperature; bpm = beats per minute; rmpm = respiratory movements per minute; mmHg = millimeters of mercury; °C = degree Celsius; mg/dL = milligram per deciliter.

## Data Availability

The original contributions presented in the study are included in the article. Further inquiries can be directed to the corresponding author.
